# Evaluating patient experiences in decentralised acute care using the Picker Patient Experience Questionnaire; methodological and clinical findings

**DOI:** 10.1186/s12913-017-2614-4

**Published:** 2017-09-29

**Authors:** Ann-Chatrin Linqvist Leonardsen, Vigdis Abrahamsen Grøndahl, Waleed Ghanima, Espen Storeheier, Anders Schönbeck, Thor-Asbjørn Løken, Nina Carine Mikkelsen Bakken, Guro Steine Letting, Réné Holst, Lars-Petter Jelsness-Jørgensen

**Affiliations:** 1 Østfold Hospital Trust, University of Oslo, P.O. Box 300, NO-1714 Grålum, Norway; 2 Østfold University College, Østfold Hospital Trust, P.O. Box 700, NO-1757 Halden, Norway; 3 Indre Østfold Kompetansesenter, Askim Municipality, Eventyrveien 2, NO-1807 Askim, Norway; 4 Intermediæravdelingen, Halden Municipality, Kjærlighetsstien 28, NO- 1781 Halden, Norway; 5 Peer Gynt Helsehus, Moss Municipality, Peer Gynts vei 86, NO- 1535 Moss, Norway; 6 Sarpsborg Municipality, P.O. Box 237, NO- 1702 Sarpsborg, Norway; 7 Fredrikstad Municipality, P.O. Box 1405, NO-1602 Fredrikstad, Norway; 8 Syddansk Universitet, Campusvej 55, DK-5230 Odense M, Denmark

**Keywords:** Decentralised, Acute healthcare, Patient experiences, Questionnaire, Self-reported health, Comorbidity, Quality, Primary care, Socio-demographics

## Abstract

**Background:**

Decentralised acute care services have, through the establishment of municipality acute wards (MAWs), been launched in Norway. The aim is to provide treatment for patients who otherwise would need hospitalisation. Currently there is a lack of studies investigating patient experiences in such services. The aims of this study were therefore to a) translate and validate the Picker Patient Experience Questionnaire (PPE-15) in Norwegian, and b) assess patient experiences in decentralised acute care, and potential factors associated with these experiences.

**Methods:**

Patients were recruited from five municipal acute wards in southeastern Norway during the period from June 2014 to June 2015. Data on socio-demographics, length of stay and comorbidity (Charlson comorbidity index (CCI)) were collected. Patients completed the Picker Patient Experience Questionnaire (PPE-15) and the EuroQOL 5-dimension, 3-level version. Convergent validity of the PPE-15 was assessed by correlation of items in PPE-15 and the Nordic Patient Experience Questionnaire (NORPEQ). A retest of the PPE-15 was performed in a subgroup of patients approximately 3 weeks after baseline assessment. Test-retest agreement was assessed with Cohens’ unweighted Kappa.

**Results:**

A total of 479 patients responded, median age 78.0 years and 41.8% men. A total of 68 patients participated in the retest. Testing of convergent validity revealed an overall weak to moderate correlation. Kappa statistics showed from fair to good test-retest agreement. Most problems were related to continuity and transition, while fewest problems were related to respect for patient preferences. A higher Charlson comorbidity score was the only variable that was negatively associated with patient experience.

**Conclusion:**

Patients reported problems in several items of the PPE-15 after discharge from decentralised acute wards. The findings from the current study may be helpful for planning ways to improve quality of care, e.g., by providing feedback to healthcare personnel or by using patient experience as a quality indicator.

## Background

The patient experience refers to how the patients, their families and other persons who participate in their care feel about the process and structure of care, as well as the outcomes of care [1]. There is a growing recognition that patients’ perspectives are essential in achieving high quality care [2, 3]. Integrating patients’ perspectives into the evaluation of healthcare delivery is important because they indicate ways to improve care, enhance strategic decision making, meet patients’ expectations, effectively manage and monitor healthcare performance, and document benchmarks for healthcare organisations [4, 5].

Due to the lack of a common definition, the measurement of patient experiences remains challenging [6]. Despite its different meanings, patient experience is often used interchangeably with terms such as patient satisfaction, perceptions or preferences [7, 8]. However, patient experiences are usually considered less subjective than patient satisfaction because patients may be satisfied with healthcare even though they have negative experiences and vice versa [9, 10]. Nevertheless, several factors have been found to influence these experiences, including age, gender, housing and employment status, ethnicity, self-rated health, multimorbidity, and care ward characteristics [11–13].

In the Nordic countries, regular assessment of patient experiences has been practiced for a long time [14]. For instance, as a part of the national quality indicators in specialist healthcare services, Norway implemented annual patient experience surveys in all hospitals in 2011 [15]. Although they are more commonly used in hospitals, municipalities are also obliged, according to the Norwegian National Health and Care Service Act, to collect patient experiences and to take these into account when planning and organising primary healthcare services [16].

To respond to future healthcare challenges, a national healthcare reform (the Coordination Reform (CR)) has been gradually implemented in Norway from 2012 to 2016 [16, 17]. One of the main objectives of the reform was to increase the total proportion of patients who accessed health services within their local community. As a direct consequence of the CR, all Norwegian municipalities were legislated to offer a 24-h acute healthcare service beginning in 2016 (Municipal Acute Wards, MAWs). Eligible patients are those who would be normally admitted to a hospital for a condition that can be managed in a general practice setting and within a timeframe of 72 h [18]. Throughout Norway, MAWs are organised differently, some being located in nursing homes, some in “houses of health, in local medical centres in relation to a casualty or a hospital. Some MAWs have employed their own doctors dedicated to the service on a 24-h basis, while other places have employed doctors only during daytime. All of the MAWs have daily doctors’ visits on weekdays. Moreover, the MAWs differ in terms of number of beds and services offered [19].

Information about patient experiences in primary healthcare, particularly in decentralised acute healthcare, are either limited or lacking [20–23]. Only two qualitative papers describing patients’ perspectives on MAWs have been published to date, finding for example that patients view MAWs as “almost a hospital”, but at the same time different with regard to person centeredness and diagnostic opportunities [24, 25]. Furthermore, the relative influence of socio-demographic variables, length of stay, self-rated health and comorbidity on these experiences remains unknown. Such knowledge could be useful when planning and improving alternatives to hospital treatment, such as MAWs.

The aims of this study were: a) to translate and validate the Picker Patient Experience Questionnaire (PPE-15) in Norwegian, and b) to assess patient experiences in decentralised acute care, and potential factors associated with these experiences.

## Methods

### Setting and participants

Østfold County, located in southeastern Norway, consists of approximately 300,000 inhabitants. The county is divided into 17 municipalities that belong to the same hospital catchment area. In addition to the hospital, a total of five MAWs have been established in different geographical locations throughout the county. The MAWs consist of four to eleven beds, dependent on the population size of their catchment area. Some laboratory services are offered in all of these facilities, while x-ray is accessible in three only.

Participants were recruited from all of the five MAWs during the period from June 2014 – June 2015 using a purposive, total population sampling method; all patients ≥18 years who had stayed in the MAW for a minimum 24 h and who were discharged alive were invited to participate.

### Data collection

Socio-demographic variables were self-reported by the patients and included the following: gender, age, civil status (married, single, widow/widower, in a relationship), housing status (living alone or not), educational level (compulsory school, upper secondary school or university), and employment status (still working or not). Information concerning the length of stay was collected from medical records.

Comorbidity was collected using the *Charlson comorbidity index (CCI)* [26]. The risk of death associated with each of 19 predefined diseases included in the CCI, is expressed as weights with values of 1, 2, 3, or 6. Summing the weights for all contributing diseases gives the CCI score for each patient. The CCI is calculated based on codes in the International Classification of Diseases (ICD-10). Information about the patients’ comorbid conditions as ICD-10 codes was collected from the National Patient Registry (NPR), which includes data on all patients treated in Norwegian government-funded hospitals.

### Questionnaire

Patient Experience was measured using the Picker Patient Experience Questionnaire (PPE-15) [27]. The final choice of questionnaire was a result of several discussions between the researchers and collaborating physicians in the community over a one-year period. Even though other translated and validated instruments existed, they were viewed either as too extensive or too short (not covering all areas of interest). The PPE-15 was developed to elicit feedback from patients to highlight aspects of care that needed improvement and to monitor performance and care. It consists of 15 questions distributed to seven dimensions of care: respect, coordination, information/communication/education, physical comfort, emotional support, involvement of relatives, and transitions and continuity [27, 28] (Appendix). The questions have two (“yes” or “no”) to four response options (“yes”,” no”, “I did not need to”, or “yes, to some extent”). Neutral answers, such as “I did not need to”, and the most positive answer are coded as a “non-problem” (score = 0). The remaining responses are coded as “problems” (score = 1). The PPE-15 has previously been found to be valid and reliable [28].

The PPE-15 has not been translated into Norwegian, and forwards and backwards translation were consequently performed according to recommendations in the literature [29, 30]: Two professional bilingual translators with Norwegian as their mother tongue performed two independent translations into Norwegian. After comparing the translations and synthesizing these into one, the questionnaire subsequently underwent a backward translation to English by a translator with English as her mother tongue. Finally, three independent individuals evaluated the questionnaire by comparing the English and Norwegian versions with regard to semantic, idiomatic, experiential, and conceptual equivalence. Following this procedure and prior to statistical testing, the Norwegian PPE-15 underwent testing of face validity. This was done by distributing the questionnaire to 10 patients prior to the study period in order to assess the adequacy, appropriateness and understandability of the questionnaire, including language and scoring instructions [31]. Patient feedback did not reveal any problematic issues in any of these aspects. Following these procedures, a final version of the PPE-15 was approved and tested.

To assess patients’ self-reported health, we used the *EuroQOL 5-dimension, 3-level version (EQ-5D-3 L)* [32, 33]. The EQ-5D-3 L consists of the EQ-5D descriptive system that measures health-related quality of life on five dimensions: mobility, self-care, usual activities, pain/discomfort, anxiety/depression, and the EQ visual analogue scale (EQ VAS). Responses are scored according to three levels: 0 (no problem), 1 (some problems) to 2 (severe problems). The EQ-5D-3 L score was used as an overall EQ-5D-3 L index score by assigning weights to each level of each dimension according to the Europe VAS value set [32, 34].

### Procedure

The following standardised inclusion procedure was used: 1) Prior to discharge, the study nurses provided the patients with oral information about the purpose of the study. 2) At discharge, the patients received written information, the study questionnaires and a consent form. 3) The patients were asked to complete these forms at home and were instructed to return the completed questionnaires, along with the signed informed consent form, in a pre-stamped envelope. Non-responders were reminded once by a phone call from the first author approximately 2 weeks after discharge.

### Statistical analysis

Summative statistics were used to present characteristics of the sample. Because the data (age, length of stay, comorbidity and self-rated health) were not normally distributed, the continuous variables are displayed as the median, mean and standard deviation. Because no method for calculating missing items exists for the PPE-15, and based on recommendations from statistical expertise at the Picker Institute (personal communication – available from the first author upon request), missing items were not included in the analysis. A Mann-Whitney U test was used to evaluate differences between responders and non-responders.

To assess convergent validity, Spearman’s correlation between of the PPE-15 items and items in the previously validated *Nordic Patient Experience Questionnaire* (NORPEQ) questionnaire was used. The NORPEQ consists of six questions that cover important aspects of healthcare encounters and is scored from the worst experience (0) to the best experience (100) [35]. We hypothesised that a generally moderate correlation would be found between the two questionnaires because the PPE-15 and NORPEQ do not capture exactly comparable aspects of the patient experience. We also hypothesised that the second item of the NORPEQ would have an overall low correlation with the PPE-15, since none of the items in the PPE-15 measure doctors’ professional skills.

Internal consistency of the PPE-15 was assessed by Cronbach’s alpha. In order to measure test-retest reliability of the PPE-15, a subgroup of responders was invited to fill out the PPE-15 a second time, approximately 3 weeks after the first completion. Invitation to participate in retest was sent consecutively as completed questionnaires were received. The literature suggests that only patients in a stable condition should be included in the re-test, since responses may be influenced by a change in health status [36]. Hence, patients were also asked to indicate whether their condition was unchanged, had deteriorated or improved. Test- retest was assessed using the Cohens’ unweighted Kappa statistics. Kappa less than 0.2 is defined as ‘poor agreement’, 0.2–0.4 as ‘fair agreement’, 0.4 to 0.6 as ‘moderate agreement’, 0.6 to 0.8 as ‘good agreement’, and a kappa =0.8 to 1.0 as ‘very good agreement’ [37].

The proportion of the PPE-15 items scored as a ‘problem’ (dependent variable, in long format) was estimated by a binomial linear mixed model [38–40] that used socio-demographic variables, length of stay, the Charlson score and the EQ-5D index score as covariates (independent variables). Some of the variation in the patients’ scores can be attributed to individual experiences as well as to aspects of the different locations (e.g., the staffing situation and the type of services they offer) [39]. The care wards and patients (identity) were consequently included as random effects to account for the inhomogeneity among patients and inhomogeneity among wards. Insignificant variables were removed from the model one at a time until only significant effects remained. The ICC was calculated to explore the proportion of random variation.

All tests were two-sided, used a 95% confidence interval (CI) and used a significance level of 0.05. All analyses were performed using Statistical Package for the Social Sciences (SPSS) version 21 [41].

## Results

During the inclusion period, 1235 (56.6%) patients, out of the 2182 patients who were admitted to the five MAWs, received a questionnaire. The proportion of discharged patients who received a questionnaire varied from 36.9% to 68.9% in the five MAWS. A total of 479 patients (38.8%) returned the questionnaires. Table 1 presents an overview of the responders’ socio-demographic characteristics, length of stay, Charlson comorbidity score and self-rated health.

**Table 1:** Study responders’ socio-demographic characteristics, length of stay, Charleson co-morbidity score and self-rated health

Male	41.8%
Mean age (SD)- in years	74.9 (14.5)
Median age- in years	78.0
Relationship (yes)	51.7%
Living alone (yes)	49%
Higher education (yes)	18.5%
Work (yes)	9%
Mean length of stay (SD)-in days	3.73(2.3)
Mean CCIS (SD)^a^	1.09 (1.6)
Mean EQ5D3L (SD)^b^	.52 (.26)

Abbreviations and table legends: *MAWs* municipality acute wards, *Male* the percentage of males in the sample, *SD* standard deviation, Relationship-married or in a relationship (Not in relationship- single or widower/widow). Higher education- high school level or above. Work- responders still working. CCIS- Charleson comorbidity index score^a^. EQ5D-EuroQol 5-dimension-3 level version index score^b^

^a^CCIS is based on nineteen predefined diseases, expressed with the values 1, 2, 3 or 6, are included in the CCI based on their association with one-year mortality. Summing the weights gives the CCI score (CCIS) for each patient

^b^Calculated with the Europe VAS score. Score range 0–1, where 0 indicates perfect health (no problems), and 1 indicates worst possible health (extreme problems on all items)

For non-responders, mean age was 78.1 years (median = 83, SD 15.2), 36.2% male. Compared with responders, non-responders were older (*p* < 0.001) and fewer were male (*p* < 0.001).

### PPE-15, validity and reliability

The correlation between the NORPEQ and the PPE-15 items varied between −0.46 and 0.43 (Table 2). The internal consistency of the PPE-15 as assessed by Cronbach’s alpha was 0.83.

**Table 2:** Correlations (Spearman Rho) between the items in the NORPEQ and the PPE-15 (*n* = 68)^a^

PPE-15 ITEMS	NORPEQ 1 “Understanding doctors”	NORPEQ 2 “Trust doctors”	NORPEQ 3 “Trust personnel”	NORPEQ 4 “Caring personnel”	NORPEQ 5 “Interested personnel”	NORPEQ 6 “Receive information”
PPE-1	−0.45**	−0.27*	−0.29*	−0.20	−0.39**	−0.34**
PPE-2	−0.32**	−0.18	−0.26*	−0.23*	−0.34**	−0.36**
PPE-3	0.23*	0.24*	0.23*	0.26*	0.28*	0.21
PPE-4	−0.19	−0.01	−0.14	−0.39**	−0.23*	−0.23
PPE-5	0.27*	0.22	0.09	0.17	0.30**	0.31**
PPE-6	0.39**	0.33**	0.18	0.27*	0.43**	0.28*
PPE-7	−0.22	−0.08	−0.28*	−0.28*	−0.20	−0.22
PPE-8	−0.09	−0.00	−0.11	−0.31**	−0.17	−0.19
PPE- 9	−0.15	−0.07	−0.19	−0.46**	−0.36**	−0.27*
PPE-10	−0.13	−0.17	−0.29*	−0.26*	−0.34**	−0.26*
PPE-11	−0.03	−0.20	−0.18	−0.29*	−0.17	−0.10
PPE-12	0.07	−0.19	−0.22	−0.33**	−0.07	−0.16
PPE-13	−0.03	−0.13	−0.31**	−0.25*	−0.11	−0.09
PPE-14	−0.05	0.04	−0.22	−0.20	−0.08	−0.12
PPE-15	−0.17	−0.05	−0.31**	−0.22	−0.31**	−0.33**

NORPEQ items: 1 = did doctors talk so that you could understand them? 2 = did you trust the doctors’ professional skills? 3 = did you trust the personells’ professional skills? 4 = did you experience that the personell cared for you? 5 = were the doctors and personell interested in your situation? 6 = did you receive information about tests and examinations? PPE-15 items as described in Appendix. Correlations as measured by the Spearman’s rho. **-significant at a 0.01 level (2-tailed). *-significant at a 0.05 level (2-tailed). The highest correlations are in bold face

^a^A positive correlation coefficient indicates a positive relationship between the two variables (the larger value PPE, the larger value NORPEQ) while a negative correlation coefficients expresses a negative relationship (the larger value PPE, the smaller value NORPEQ)

Cohens’ unweighted Kappa statistics varied from 0.27 (fair agreement) - 0.7 (good agreement), except from on PPE-15 item 11, in which the agreement was 0.13 (poor) (Table 3).

**Table 3:** Test-retest of the PPE-15 using unweighted Kappa coefficient (n = 68)

PPE-15 item	Kappa	*p*-value
PPE-1	0.431	<.001
PPE-2	0.538	<.001
PPE-3	0.431	<.001
PPE-4	0.447	<.001
PPE-5	0.409	<.001
PPE-6	0.562	<.001
PPE-7	0.698	<.001
PPE-8	0.297	.012
PPE-9	0.400	.001
PPE-10	0.597	<.001
PPE-10a	0.473	<.001
PPE-11	0.125	.275
PPE-12	0.311	.008
PPE-13	0.266	.018
PPE-14	0.479	<.001
PPE-15	0.357	.002

Abbreviations: *PPE* Picker Patient Experience Questionnaire-15 items as described in the Appendix

### Patient experiences and association with background characteristics

The number of respondents who reported each PPE-15 item as a problem and the item’s corresponding dimension are presented in Table 4. Fourteen of the respondents did not report any problems.

**Table 4:** Proportion of responders reporting problems on the items of Picker Patient Experience Questionnaire (PPE-15)

PPE-15 item	*n* = 479
1) Understandable answers to questions from doctors [70]	118/475 (24.8)
2) Understandable answers to questions from nurses [70]	114/475 (24)
3) Different answers from different personnel [70]	148/475 (31.2)
4) Discuss anxieties/fears about condition/treatment with doctor (3)	214/475 (45.1)
5) Doctors talk in front of you, as if you weren’t there? (4)	42/475 (8.8)
6) Involvement in care and treatment decisions? (4)	200/475 (42.1)
7) Treated with respect and dignity (4)	42/476 (8.8)
8) Discuss anxieties/ fears about condition/treatment with nurse (3)	146/476 (30.7)
9) Someone in staff to talk to about concerns? (3)	142/475 (29.9)
10) Were you ever in pain? (yes)	347/475 (73.1)
10a) Staff took action to relieve pain (5)	99/476 (20.8)
11) Opportunity for family/close persons to talk to doctor (6)	103/477 (21.6)
12) Enough information to family or someone close to help recover? (6)	139/476 (29.2)
13) Understandable explanation about the purpose of medicines (7)	121/475 (25.5)
14) Information about medication side effects (7)	243/475 (51.2)
15) Information about danger signals to observe at home (7)	348/475 (73.3)

Abbreviations and table legends: *MAWs* municipal acute wards, *PPE*-15-Picker Patient

Experience Questionnaire. PPE-15 item-the 15 items, with dimension 1–7 in parenthesis; 1 = Information and education, 2 = Coordination of care, 3 = Emotional comfort, 4 = Respect patient preferences, 5 = Physical comfort, 6 = Involvement of family and friends, 7 = Continuity and transition. PPE-15 item- Picker Patient Experience Questionnaire, short version of the 15 questions. The proportion of responders reporting a problem on number of answers to each of the PPE-15 items. Percentage in parenthesis

The largest proportion of problems was observed in the continuity and transition dimension of the PPE-15, particularly the item related to information about dangerous signals to watch for at home (73.3%). The smallest proportion of problems were observed for the item related to being treated with respect and dignity, and the item asking whether doctors talked in front of them as if they were not there (8.8%, respectively). Furthermore, 42.1% of patients reported problems related to their involvement in treatment and care. While 25.5% of patients reported problems related to whether explanations about the purpose of medicines were understandable, and 51.2% reported problems related to information about medication side effects.

The random effects analysis revealed a negligible variation among wards (ICC < 0.001), whereas the random variation between patients within wards contributed to 21% of the total random variation. In the binominal linear mixed model only the Charlson comorbidity score was statistically significantly associated with decreased patient experience, while factors such as gender, age, self-rated health, length of stay, educational background, employment status, housing status and civil status were not associated with patient experiences (Table 5).

**Table 5:** Results from the binomial linear mixed model of the PPE-15, using care wards (*n* = 5) and patients (n = 479) as random effects

	OR	CI (95%)	*p*-value
Civil status	1.001	(0.581–1.726)	.997
Educational background	0.988	(0.788–1.255)	.922
Housing status	1.020	(0.841–1.237)	.839
EQ5D3L	1.050	(0.789–1.397)	.738
Employment status	0.670	(0.066–6.774)	.734
Gender	0.982	(0.773–1.113)	.420
Age	1.004	(0.997–1.010)	.259
Length of stay	1.045	(1.009–1.081)	.13
CCIS	1.085	(1.011–1.164)	.023

Abbreviations and table legends: *OR* odds ratio, *CI* confidence interval, *EQ5D3L* EuroQol 5-dimension-3 level version index score, *CCIS* Charleson comorbidity index score. Insignificant variables were removed from the model one at a time until only significant effects remained. The OR, CI and *P*-values presented in this table is the value of each factor prior to being omitted in the step-wise analysis (please see methods)

## Discussion

Findings indicate that the Norwegian PPE-15 is a valid and reliable patient experience questionnaire. Furthermore, among patients admitted to MAWs, the highest proportion of problems were related to aspects of ‘continuity and transition’. Increased comorbidity was the only factor that was significantly negatively associated with patients’ experiences in this study.

### PPE-15, validity and reliability

The PPE-15 has primarily been tested in hospitalised patients [28]. The dimensional structure is based on input from patients during it’s development and not on any statistical tests, such as e.g., factor analysis [27]. The latter might be because no dimensional score exist. Consequently, the dimensions reported only reflect what items are perceived by patients to be semantically related. Similar testing have been performed in Sweden [42]. As a consequence of the procedures used in prior studies and scoring instructions, a factor analysis was not used in the current study. However, during the assessment of face validity, patients did not report any specific issues related to neither the content of PPE-15 nor the items corresponding dimensions.

Testing of convergent validity revealed an overall weak to moderate correlation between the NORPEQ and the PPE-15 items. This may be because the specific content of each item differs between these two questionnaires. However, correlations were generally higher in those items hypothesised to be more closely associated, and lower in those not hypothesised as associated. This may indicate that the PPE-15 has an acceptable convergent and discriminant validity.

The Kappa values varied from fair to good agreement in the vast majority of items. In item 11, concerning whether family and close ones had the possibility to talk to a doctor if they were in need or wanted to, the agreement was poor. The potential reason for this finding is unclear and future studies using the PPE-15 should explore this more closely.

### Patient experiences

Our results show that the vast majority of patients reported that they were treated with respect and dignity, which corresponds with findings from a qualitative study [25]. Nevertheless, many wanted greater involvement in decisions about their care and treatment. Although this finding is in line with a study of 34,000 hospitalised patients in Sweden [43], our results seem to contrast prior reports that have observed a positive association between respect/dignity and involvement in care [44]. Patient-centred care has been described as a partnership between patients and healthcare professionals to inform and involve patients in shared decision making [45]. Outcomes have been suggested to include patients’ feeling of respect, involvement, engagement and knowledge [46].

Patients in the current study also experienced problems related to continuity and transition. Prior studies have shown that poor communication, incomplete transfer of information, and inadequate education of the patients may have a negative impact on care transitions, leading to unplanned readmissions and adverse events [47, 48]. In particular, problems were reported concerning information about potential medication side effects to observe at home. Drug-related problems (DLPs) occur quite frequently after discharge, and factors such as a short stay and inadequate communication may influence the figure negatively [49]. Studies have however found that providing elderly patients with medication reports may reduce this amount [49, 50]. Interestingly, 24–24.8% of patients in our study reported problems related to receiving understandable answers to questions from either nurses or doctors, while 31.2% reported that they had received different answers to questions from different personnel. Studies have found that significant differences exist between what physicians think patients know and what patients actually know [51]. Because we do not have any specific information on how the discharge process was handled at each treatment location, it is beyond the scope of this paper to draw any firm conclusions in that regard. Nevertheless, the variation among care wards was negligible in the current study. However, given the importance of discharges and handovers, it seems important to target quality of patient and healthcare personnel communication to improve how patients experience their healthcare encounter [52].

The results from our study show that the random effect attributed to the ward to which patients were admitted had very little influence on patient experiences. This may be due to a relatively small sample size, or that participants were included from one county were the MAW routines were quite homogenous, due to joint collaborative efforts. Interestingly, prior studies have argued that for most quality aspects, including the ward into analyses of patient experiences is important for a number of quality indicators [53].

### Factors influencing patient experiences

Our findings show that age, gender, civil status, employment status, educational status, housing status, self-rated health and length of stay had no significant effect on the number of problems. This is in contrast to earlier research reporting that increased age and decreased self-reported health status are the strongest predictors of negative patient experiences [54]. Additionally, one study found that at least 79% of the variance of all patient experience measures occurred at the patient level [9]. In a study of 34,000 Swedish hospitalised patients, poorer experiences were associated with greater healthcare utilisation, higher age, functional impairment and female gender. Previous studies have also suggested a positive association between older age and higher care satisfaction [54, 55]. However, studies on primary care services have also been inconsistent regarding the relative influence of health and socio-economic status, age, gender, ethnicity and self-rated health on patient reported experiences [56, 57]. It might be speculated if our findings occurred because the PPE-15 uses a dichotomised score.

A greater number of comorbid conditions was weakly, but significantly negatively associated with patient experiences. Comorbidity is associated with worse health outcomes, more complex clinical management, and increased health care costs [58]. In older adults, comorbidity is one of three factors (along with frailty and disability) that are commonly used to indicate vulnerability [59]. Vulnerable patients have also reported fewer positive care experiences than non-vulnerable patients in prior studies [43]. Healthcare professionals may view vulnerable patients as individuals who have more complex healthcare needs; thus, they may focus on meeting these needs rather than focusing on patient satisfaction or patient experiences [43].

### Limitations

Increasing time since discharge seems to result in poorer patient experiences scores, and patients who self-report their experiences at home following discharge may consequently have an increased risk of recall bias [60]. While collecting data before discharge from MAWs could have increased the number of respondents and reduced the risk of recall bias, the responses could also have been influenced by potential interruptions and influence of the personnel [61].

Sadly, not all patients who were discharged from the five MAWs were invited to participate, as planned. This could indicate a potential selection bias. Based on input from the various MAWs and study follow-up, there did not seem to be any pattern regarding who was invited and who was not. The reasons seemed to be lack of time due to many tasks, and forgetfulness. Another drawback is that we did not collect information on those who did not receive a questionnaire, which of course makes it impossible to assess any differences between those who were invited and those who were not. Moreover, the response rate is relatively low. It is therefore difficult to know if those not responding had worse experiences, which has been reported in the literature [62]. Comparison of non-responders and responders furthermore revealed significant differences in gender and age.

In retrospect, we could have invited a larger number of patients in the retest, yet the number of patients needed in these tests have been subject of debate. For instance some have advocated that a sample size of 50 could be sufficient or a starting point, while others have highlighted the need for larger sample sizes and more robust test-retest data [63, 64]. Indeed, a systematic review found that the median number included in retest analysis was 60 [36].

Patients may judge an organisation in light of personal expectations, and this may not adequately reflect actual care quality. Pre-existing higher expectations of care quality have been linked to increased critical evaluation of a healthcare service [65]. Our study did not include questions about patient expectations, and consequently, we cannot conclude whether worse experiences reflect suboptimal care or different expectations [66]. However, because MAWs are newly established health services, patients may not adequately know what to expect when they are treated in these units [24].

## Conclusion

The PPE-15 displayed acceptable validity and reliability. While patients reported problems in all of the PPE-15 items, this was particularly evident in aspects related to discharge information. These findings may be helpful in planning ways to improve quality of care, e.g., by providing feedback to healthcare personnel, or by using patient experience as a quality indicator. Findings may also be important when developing new decentralised acute health care services.

## Appendix

**Table 6:** Domains, items and scoring alternatives of the Picker Patient Experience Questionnaire

Domain and Item	Scoring alternatives
Information and education	
Item 1 - When you had important questions to ask a doctor, did you get answers that you could understand?	Yes, always/Yes, sometimes/No/I had no need to ask
Item 2 - When you had important questions to ask a nurse, did you get answers that you could understand?	Yes, always/Yes, sometimes/No/I had no need to ask
Coordination of care	
Item 3 - Sometimes in a hospital, one doctor or nurse will say one thing and another will say something quite different. Did this happen to you?	Yes, often/Yes, sometimes/No
Emotional comfort	
Item 4 - If you had any anxieties or fears about your condition or treatment, did a doctor discuss them with you?	Yes, completely/Yes, to some extent/No/I didn’t have any anxieties or fears
Item 8 - If you had any anxieties or fears about your condition or treatment, did a nurse discuss them with you?	Yes, completely/Yes, to some extent/No/I didn’t have any anxieties or fears
Item 9 - Did you find someone on the hospital staff to talk to about your concerns?	Yes, definitely/Yes, to some extent/No/I had no concerns
Respect patient preferences	
Item 5 - Did doctors talk in front of you as if you weren’t there?	Yes, often/Yes sometimes/No
Item 6 - Did you want to be more involved in decisions made about your care and treatment?	Yes, definitely/Yes, to some extent/No
Item 7 - Overall, did you feel you were treated with respect and dignity while you were in hospital?	Yes, always/Yes, sometimes/No
Physical comfort	
*Item 10 - Were you ever in pain?*	*Yes/No*
Item 10 a - If yes… Do you think the hospital staff did everything they could to help control your pain?	Yes, definitely/Yes, to some extent/No
Involvement of family and friends	
Item 11 - If your family or someone else close to you wanted to talk to a doctor, did they have enough opportunity to do so?	Yes, definitely/Yes, to some extent/No/No family or friends were involved/My family didn’t want or need information/I didn’t want my family or friends to talk to a doctor
Item 12 - Did the doctors or nurses give your family or someone close to you all the information they needed to help you recover?	Yes, definitely/Yes, to some extent/No/No family or friends were involved/My family or friends didn’t want or need information
Continuity and transition	
Item 13 - Did a member of staff explain the purpose of the medicines you were to take at home in a way you could understand?	Yes, completely/Yes, to some extent/No/I didn’t need an explanation/I had no medicines—go to question 15
Item 14 - Did a member of staff tell you about medication side effects to watch for when you went home?	Yes, completely/Yes, to some extent/No/I didn’t need an explanation
Item 15 - Did someone tell you about danger signals regarding your illness or treatment to watch for after you went home?	Yes, completely/Yes, to some extent/No

## Abbreviations

CCIS: Charleson comorbidity index score
EQ5D: EuroQol 5-dimension-3 level version index score
MAW: Municipality acute ward
PPE-15: Picker Patient Experience Questionnaire
